# Cervical Epidural Abscess Secondary to a Post-Traumatic Hematoma, Successfully Treated with Adjunctive Hyperbaric Oxygen Therapy: A Case Report

**DOI:** 10.3390/jcm14207346

**Published:** 2025-10-17

**Authors:** Yoshiaki Iwashita, Naho Yoshioka, Kotaro Murakami, Ken Mukoyama, Rie Sato, Nobuhiro Kodani, Tetsuya Makiishi

**Affiliations:** 1 Department of Emergency and Critical Care Medicine, School of Medicine, Shimane University, Izumo 693-8501, Shimane, Japan; 2 Department of General Medicine, Faculty of Medicine, Shimane University, Izumo 693-8501, Shimane, Japan

**Keywords:** spinal epidural abscess, hyperbaric oxygen therapy, spinal epidural hematoma

## Abstract

**Background:** Spinal epidural abscess (SEA) is a rare but challenging disease. Hyperbaric oxygen therapy (HBOT) is used as an adjunctive therapy for SEA in a limited number of hospitals; however, its efficacy has not been well described. **Case:** A 70-year-old man presented at our hospital with cervical pain, fever, and impaired right shoulder movement. The patient fell after drinking 17 days prior to the presentation. He was diagnosed with an SEA secondary to a spinal epidural hematoma caused by a prior injury. The patient also had dental caries, and his blood culture was positive for *Streptococcus intermedius*. We diagnosed the patient with a spinal epidural abscess and hematoma that developed from the caries and the injury. Antibiotics were initiated; however, the motor function gradually worsened, and decompressive surgery was performed. After surgery, neurological impairment persisted, and HBOT was used as an adjunctive therapy. After initiating HBOT, the patient’s arm movements improved, and he was referred to a rehabilitation hospital on day 110 for further rehabilitation. **Conclusions:** HBOT is increasingly used for spinal cord infections and injuries in a limited number of institutions. It is a potentially effective adjunctive therapy for patients for whom antibiotics and surgery are ineffective.

## 1. Introduction

Spinal epidural abscess (SEA) is an uncommon but serious infection of the spinal canal with an estimated incidence of 0.2–2.0 cases per 10,000 hospital admissions, and its prevalence has been increasing in parallel with aging populations and the wider use of spinal instrumentation and invasive procedures [1,2]. SEA occurs more frequently in patients with diabetes, chronic renal failure, immunosuppression, and intravenous drug use, but may also develop in otherwise healthy individuals. While most cases are non-traumatic in origin, secondary SEA following trauma or spinal epidural hematoma remains rare but has been reported [3,4]. The condition predominantly affects middle-aged to elderly adults, with a slight male predominance, and delays in diagnosis are strongly associated with worse neurological outcomes [1,2].

Standard therapy consists of prolonged intravenous antibiotics, commonly for over 6 weeks, combined with surgical drainage when indicated. Despite appropriate treatment, SEA can still result in permanent neurological deficits if diagnosis or intervention is delayed. Adjunctive therapies that can enhance infection control and neurological recovery are therefore of clinical interest.

Hyperbaric oxygen therapy (HBOT) is one such adjunct. The Undersea and Hyperbaric Medical Society (UHMS) currently recognizes fourteen established indications for HBOT, including intracranial abscess and refractory osteomyelitis, both of which share pathophysiological similarities with SEA [5]. The proposed mechanisms of HBOT in SEA include increased tissue oxygenation, restoration of oxygen-dependent leukocyte bactericidal activity, improved antibiotic efficacy, reduced edema, and promotion of angiogenesis and wound healing [5]. Although SEA is not among the standard UHMS-approved indications, a growing number of case reports and small series have described its successful use as an adjunct in refractory or high-risk SEA cases.

Here, we present a rare case of SEA secondary to a spinal hematoma that was successfully treated with HBOT in combination with standard therapy.

## 2. Case Presentation

A 70-year-old man fell forward at home after alcohol intake and developed neck and shoulder pain 17 days prior to presentation to our hospital. On day 0, he presented with severe neck pain, inability to abduct the right shoulder or flex or extend the elbow, and reduced oral intake. Upon examination, the patient’s temperature was 39.0 °C, with a heart rate of 132 bpm, blood pressure of 113/63 mmHg, and oxygen saturation of 98% on room air. Neurologically, right upper limb weakness was present (Manual Muscle Test (MMT): shoulder joint abduction 0/1, elbow flexion 3/3, elbow extension 4/4, wrist flexion 5/5, wrist extension 5/5, finger flexion 5/5, and finger extension 4/4), whereas lower limb function was intact.

**Figure 1:**
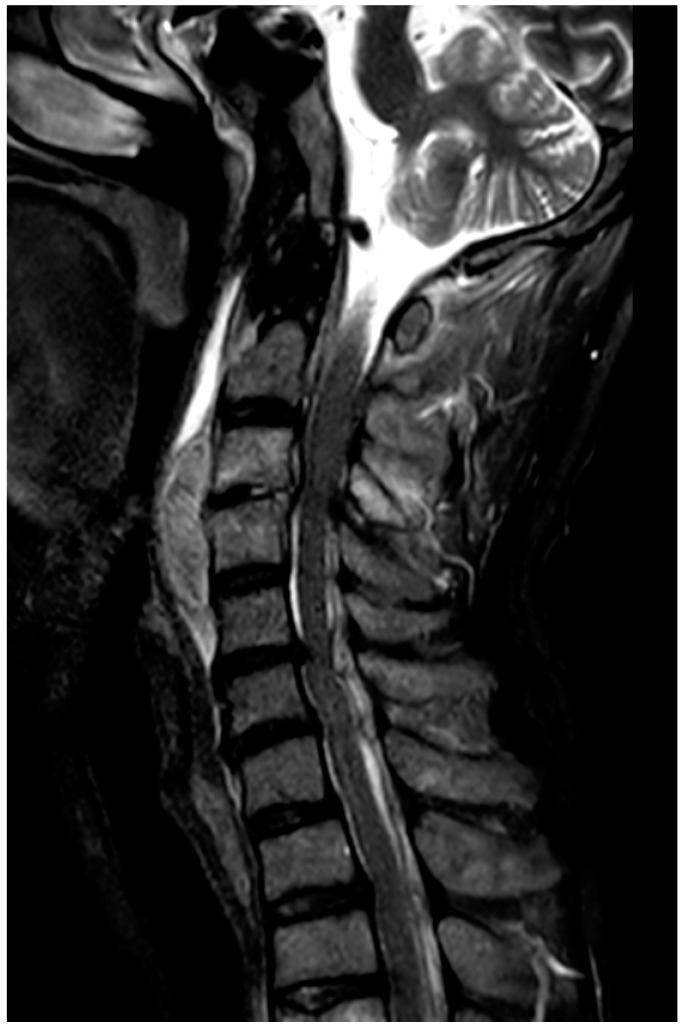
MRI T2 STIR. Mid sagittal STIR MRI on admission (day 0) showing a dorsal epidural lesion at C3–C5 compressing the spinal cord; high signal with rim enhancement consistent with secondary infection of a post-traumatic hematoma.

Laboratory data showed C reactive protein levels of 31.5 mg/dL and procalcitonin levels of 1.57 ng/mL with anemia and thrombocytopenia. Computed tomography (CT) and magnetic resonance imaging (MRI) of the cervical spine (Figure 1) revealed a C3 fracture with ventral and dorsal epidural space-occupying lesions compressing the cord.

**Figure 2:**
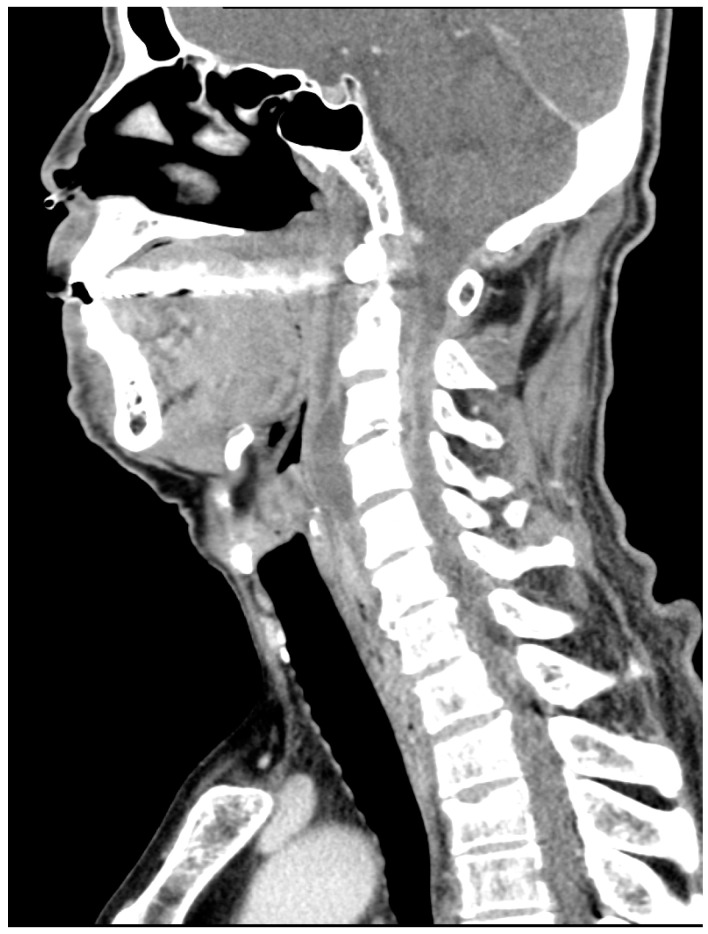
Enhanced cervical CT on admission (day 0) showing a low-density area with a peripheral enhancement effect. Vertebral body and adjacent soft tissue suggest C3/4 spondylosis and an abscess formation.

On subsequent contrast-enhanced CT (Figure 2), the collection exhibited peripheral rim enhancement with a progressive mass effect, consistent with superinfection of the pre-existing hematoma.

Blood cultures yielded *Streptococcus intermedius*, and dental assessment revealed multiple untreated caries, supporting a dental source. Taken together, SEA appeared to have arisen from a secondary infection of a post-traumatic epidural hematoma caused by bacteremia due to a dental infection. No other foci of infection, including infective endocarditis, were observed.

At the time of admission, imaging studies showed that compression of the dural sac was not severe. Since more than 14 days had passed since the injury and spontaneous absorption of the hematoma were expected, surgical intervention was not considered, as indicated. Empirical intravenous antibiotic treatment with meropenem was initiated. The antibiotic regimen was changed to ampicillin on day 6, when the results of blood cultures revealed the antimicrobial susceptibility profile. Fever and inflammation decreased after starting antibiotics, but no remarkable changes in neurological symptoms were observed. On day 9, MMT worsened to shoulder joint abduction 0/1, elbow flexion 0/2, elbow extension 2/2, wrist flexion 2/2, wrist extension 2/2, finger flexion 2/3, and finger extension 2/2, and surgery was performed to decompress and drain the spinal cord. The surgical procedure was C5–6 laminoplasty, partial laminectomy of C2 and C7, and complete laminectomy of C3 and C4. The neurological findings on the day after surgery were the same as those observed before surgery. Despite surgery and culture-directed antibiotics, the clinical symptoms remained unchanged.

**Figure 3:**
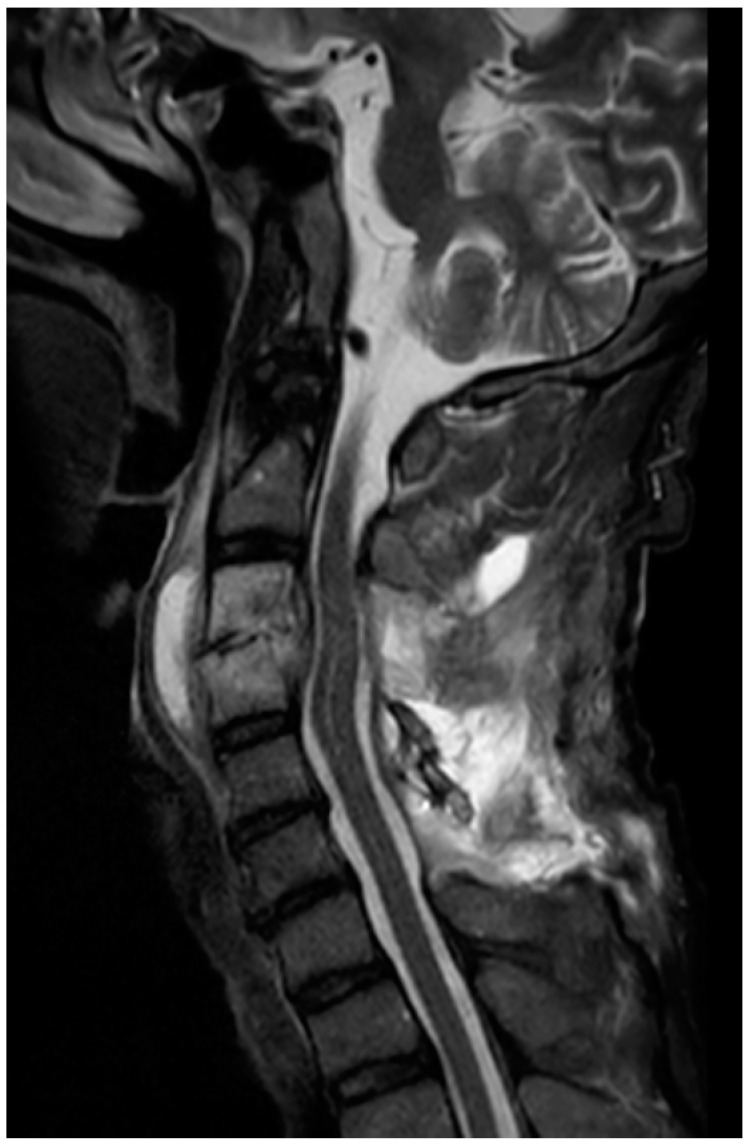
MRI T2 STIR on day 23. Although anterior compression at the C3–4 level has progressed, spinal canal stenosis has not developed because laminectomy had been performed.

Adjunctive HBOT was initiated on day 14 (post-operative day 5), with a treatment protocol of 2.0 atm. absolute (ATA) for 60 min once daily for a total of 30 sessions. The regimen was under the recommendation of The Japanese Society of Hyperbaric and Undersea Medicine. Following HBOT, pain improved, inflammatory marker levels decreased, and motor function recovered. On day 23, MMT improved to shoulder joint abduction 1/2, elbow flexion 2/3, elbow extension 4/3, wrist flexion 4/4, wrist extension 3/3, finger flexion 4/4, and finger extension 4/4. MRI on day 23 showed anterior compression of the spinal cord at the C3–4, but spinal canal stenosis had not developed because laminectomy had been performed (Figure 3).

An anterior approach for surgical intervention was considered to address the abscess located at the anterior aspect of the vertebral body. However, since anterior fixation might be required later following the laminectomy, we chose to avoid multiple surgical procedures. In addition, because inflammatory markers remained low with antibiotic therapy, we decided to continue conservative management. MRI on day 89 demonstrated a reduction in epidural collection, cord decompression and pre-vertebral abscess. Intravenous antibiotics were continued until day 91 and then changed to oral antibiotics. On day 110, the patient regained right upper limb function and was transferred to a rehabilitation hospital.

## 3. Discussion

We report a rare case of SEA secondary to a traumatic epidural hematoma that was successfully treated with antibiotics, surgery, and adjunctive HBOT. HBOT is used for the treatment of SEA in certain hospitals; however, it is still not considered a standard treatment. The standard treatment for SEA is still a combination of antibiotics and surgery; however, symptoms in this case did not resolve with standard treatment, and adjunctive HBOT seemed to enhance treatment efficacy.

The basis of SEA management is urgent surgical decompression when neurological deficits are identified [1,2]. A few reports have described SEA with *Streptococcus intermedius* due to dental infections [6,7]. Although standard therapy has been described, some patients demonstrate suboptimal recovery despite receiving appropriate therapy. Reports indicate poor neurological outcomes when decompression is delayed beyond 24–36 h, even when antibiotics are administered [8]. Delayed diagnosis and treatment remain critical determinants of functional prognosis. In our patient, more than two weeks had elapsed since the initial trauma, and the MRI at admission showed only mild dural sac compression; therefore, conservative management with antibiotics was initially selected. Surgery was subsequently performed when infection was confirmed and neurological deficits progressed. However, neurological improvement was not observed immediately after surgery. HBOT was initiated on postoperative day 5, corresponding to day 14 after admission, as an adjunctive therapy to enhance spinal cord oxygenation and facilitate recovery.

HBOT increases dissolved oxygen and tissue oxygen tension, augments phagocytic killing, reduces edema, and potentiates the efficacy and penetration of selected antibiotics into hypoxic or poorly perfused compartments [5]. Although SEA is not a routine indication in major HBOT guidance documents, several case reports have described neurological and radiological improvements when HBOT is combined with surgery and antibiotics, including a cervical SEA response within days of HBOT initiation and complex epidural abscesses managed with debridement, fixation, and adjuvant HBOT [9,10,11]. Our protocol (2.0 ATA, 60 min, 30 sessions) aligns with commonly used regimens for refractory infections. Beyond infection control, HBOT has also been explored in traumatic spinal cord injury models, where it reduces neuroinflammation, limits apoptosis, and promotes neurological recovery [12,13,14]. A systematic review suggested improvements in ASIA motor and sensory scores and functional indices following HBOT compared to standard therapy alone [13]. These findings imply that HBOT may mitigate secondary injury caused by compression and ischemia by offering neuroprotective benefits beyond its antimicrobial adjuvant role.

Secondary infection with a pre-existing epidural hematoma is rare, but mechanistically plausible. Trauma may predispose up to one-third of cases to SEA and is postulated to create a hematoma that becomes a nutrient-rich nidus under bacteremia [3,4]. We acknowledge certain limitations in our management. The delay in surgical intervention may have contributed to the initial severity and persistence of neurological deficits. Moreover, HBOT was introduced after surgery; earlier initiation might have facilitated faster or more complete recovery, although this remains speculative. Causality could not be proven in a single case, and the delayed effects of surgery or antibiotics could not be excluded. However, the temporal association between HBOT initiation and clinical infection supports this contributory role. HBOT should not delay urgent surgery or antimicrobial therapy; however, our findings demonstrate that it can be considered as an adjunctive therapy, though further studies are warranted to clarify these indications.

## 4. Conclusions

In SEA arising from a secondary infection of a posttraumatic epidural hematoma due to an odontogenic source, adjunctive HBOT may aid in recovery when standard therapy yields suboptimal improvement. Early recognition of dental foci, timely decompression, and appropriate antibiotics remain essential. HBOT can be considered a non-delayed adjunctive treatment in selected cases.

## Data Availability

The additional patient data can be obtained from the corresponding author upon reasonable request, as this was a single-case report.

## Abbreviations

The following abbreviations are used in this manuscript:

SEA	Spinal epidural abscess
HBOT	Hyperbaric Oxygen Therapy
